# Water Supply

**Published:** 1911-03-25

**Authors:** 


### CONSTRUCTION AND ADMINISTRATION.

### WATER-SUPPLY.

### II.—CREATION OF SUPPLY.

Having described the leading types of well, the next point to consider is the method of creating supply for ordinary use, but it may here be said that information of a general nature regarding geological formations can be found at the National Geological Museum, Jermyn Street, London, W.

The walls of the well may be built of brick, stone or concrete, but whatever material is used care must be taken to exclude other doubtful sources of supply, such as subsoil water. This may be effected by clay puddling or by maintaining a protected area around the well for a certain depth. The well-lining should be watertight as a preventive to the percolation of subsoil water or shallow springs, and the lining should stand between 2 and 3 feet above the ground, being domed or flagged over to prevent the entrance of vegetable matter, such as leaves, and further to keep out rats or other animals which might poison the water.

Near to towns cylinders of cast or wrought iron forced down by jacks or hydraulic pressure may be used instead of brick, stone, or concrete walls; but for rural districts, where cartage and special plant would increase the outlay to a considerable amount, such methods are too costly. It is as cheap to sink a well of 4 feet diameter as a smaller one, for the workman can move with greater ease. The method of building a well of this type is that the ground is excavated as far as possible without incurring risks of collapse. An elmwood curb cut circular on the inside, but not on the outside, is then placed on the floor of the excavation, and brickwork in hydraulic lime or cement is built thereon. The excavation is then carried further, within the line of the circular elm curb, and a solid timber flooring piece placed on the ground in the middle. From this raking shores or struts are put to support the elm curb. The earth is then cut away at points under the curb and brick piers resting in their turn on a second elm curb are built ; then more earth is removed, and so on until the whole circle is complete, the elm curbs being kept in all cases in their original position. This same performance is again carried out until put a stop to by getting into the water-bearing stratum. If work then becomes impossible it is better to stop and return at a later period after the well has had time to clear and develop its water-level. The lower parts of the walls within the water-level may be built dry, *i.e.* without lime or cement to enable the water to percolate through the open joints.

Wells of the type described in our previous article may be found in all villages, and the water is sometimes raised by bucket and windlass, or by a suction tube pump, but with more modern ideas of construction the old-fashioned methods are becoming obsolete, and are described by authorities as dangerous owng to the possible contamination by organic matters or by the water bearing organic and decaying vegetable matter.

The type of well now most generally recognised, particularly in London, is the tube well, sometimes called the Norton or Abyssinian, having first come into public notice during the campaign from which it derives its name. The tubing and other apparatus for sinking such wells now form part of the Army ordinary field equipment, and are specially useful for military purposes on account of the ease and rapidity with which the tubes can be driven and withdrawn repeatedly in a free soil. The diameter of the tubes varies from 1¼ inch to 4 inches internally, the soils most favourable to their use being coarse sands and gravel, such as is found under London. The simplest type of plant for this class of well consists of 1¼-inch steam barrel shod with a steel point, and perforated with numerous small holes immediately over the steel point. As the tube is forced into the ground another length is screwed into its upper end until the desired depth is reached. This is then connected to a syphon suction pump of either centrifugal or lever pattern, depending on the amount of work required of it. The cost of a pump of this kind would be between £10 and £20, according to the number and the length of the tubes sunk. In cases, however, where the supply required is much greater, and where it would be desirable to instal an engine or motor to do the labour, as is now common in London, the cost would be considerably greater.

Whatever type of well is sunk, it is imperative that the water be properly analysed to ensure its suitability for drinking purposes.

*(To be continued.)*

